# Transthoracic Color Doppler Ultrasound-Guided Grooved Negative Pressure Drainage Tube Implantation in Pericardial Effusion After Cardiac Surgery

**DOI:** 10.21470/1678-9741-2022-0044

**Published:** 2023-08-07

**Authors:** Can Feng, Zhengwen Lei, Peng Xiyang

**Affiliations:** 1 Department of Cardiothoracic Surgery, The First Affiliated Hospital of University of South China, Hengyang, Hunan, People’s Republic of China

**Keywords:** Pericardial Effusion, Cardiac Tamponade, Color Ultrasound Doppler, Drainage

## Abstract

**Introduction:**

Pericardial effusion is a common complication without a standard postoperative effusion treatment after cardiac surgery. The grooved negative pressure drainage tube has many advantages as the emerging alternative for drainage of pericardial effusion, such as it changes the structure of the traditional side hole, uses the capillary function to ensure drainage smooth, etc. The purpose of this study was to assess the feasibility and effectiveness of transthoracic color Doppler ultrasound-guided grooved negative pressure drainage tube implantation in pericardial effusion after cardiac surgery.

**Methods:**

All patients with pericardial effusion after cardiac surgery who underwent transthoracic color Doppler ultrasound-guided grooved negative pressure drainage tube implantation between January 2019 and December 2021 were retrospectively analyzed. Treatment results (including clinical symptoms, effusion volume, color Doppler ultrasonography, and computed tomography scan) were investigated to evaluate the effectiveness and safety of this method.

**Results:**

A total of 20 patients successfully underwent transthoracic color Doppler ultrasound-guided grooved negative pressure drainage tube implantation. After the operation, their symptoms (chest tightness, shortness of breath, etc.) were all relieved, and dark red or light red drainage fluid (> 200 ml) appeared in the newly placed drainage bottle. Color Doppler ultrasonography showed that the volume of pericardial effusion decreased significantly.

**Conclusion:**

The transthoracic color Doppler ultrasound-guided grooved negative pressure drainage tube is a safe and effective method for the treatment of postoperative pericardial effusion with less trauma, faster recovery, shorter in-hospital stay, and fewer complications.

## INTRODUCTION

Pericardial effusion is one of the most common and challenging complications after cardiac surgery. A massive pericardial effusion has a life-threatening risk of progression to cardiac tamponade. The incidence of pericardial effusion after cardiac surgery is reported to be 1% ~ 77%^[1,2,3]^; most of these cases are mild or moderate, but 1% ~ 2% of them require close monitoring and intervention^[1,4]^. Therefore, timely and effective prevention and treatment of postoperative pericardial effusion has important clinical significance. At present, the treatment of pericardial effusion after cardiac surgery is mainly as follows: 1) medical medication focused on cardiotonic and/ or diuretic drugs; 2) percutaneous pericardial catheter drainage guided by color Doppler echocardiography^[4,5,6]^; 3) pericardial fenestration^[3,5,10,11,12]^. Although these methods are widely used in clinical practice, there are some limitations. So, it is necessary to develop a minimally invasive, safe, and effective pericardial drainage method for the treatment of postoperative pericardial effusion. Based on the abovementioned problems and combined with clinical experience, we summarized the experience in the treatment or prevention of postoperative pericardial effusion with a disposable sterile negative pressure groove drainage device guided by transthoracic color Doppler ultrasound.

In our medical center, transthoracic color Doppler echocardiography is performed routinely in patients after cardiac surgery before removal of the pericardium and mediastinal drainage tube^[8]^, which can not only improve the recovery of cardiac function, but also determine the pericardial effusion in order to avoid pericardial effusion retention after extubation. When the drainage is not smooth or accurate, we also routinely perform color Doppler echocardiography or chest computed tomography. If the initial characterization of the effusion is clearly indicated, we routinely use color Doppler ultrasound guidance and negative pressure groove drainage tube implantation; the drainage tube under the guidance of the guide wire is inserted into the initial position for full drainage.

## METHODS

### Patient Selection

A total of 20 patients (male:female = 3:1) with symptomatic postoperative pericardial effusion and who underwent cardiac surgery with cardiopulmonary bypass between January 1, 2019 to December 31, 2021 in the First Affiliated Hospital of University of South China were enrolled into this study (Table 1). The obvious clinical symptoms and the medical indication for therapeutic pericardiocentesis in all patients were confirmed by color Doppler ultrasound. All the procedures of this study were approved by the local ethics committee of First Affiliated Hospital of University of South China (No. 201662661218), and informed consent was signed by the patients and their families.

**Table 1:** Summary of the patients’ demographic information before operation.

Variables	Data
Sex, female, n (%)	5 (25.0)
Age (years)±SD	49.1±16.8
Body mass (kg)±SD	63.3±13.2
Classification of cardiac function (NYHA class), n (%)	
I-II	9 (45.0)
III-IV	11 (55.0)
Preoperative EF (%)±SD	45.1±27.1
Preoperative LVEDV (mm)±SD	39.6±24.1

EF=ejection fraction; LVEDV=left ventricular end-diastolic volume; NYHA=New York Heart Association; SD=standard deviation

### Transthoracic Color Doppler Ultrasound-Guided Grooved Negative Pressure Drainage (Video 1)

**Video 1:**
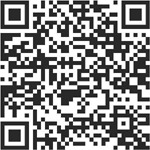
Guide wire intervention Grooved Negative Pressure Drainage.

**Fig. 1:**
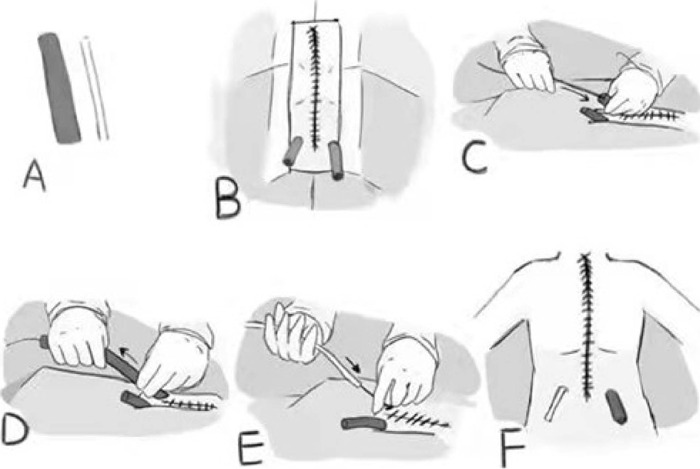
Diagram of the drainage procedure. (A) The left side is a conventional chest and pericardial drainage tube, the right side is a negative pressure groove drainage device catheter. (B) Conventional draping was placed. (C) A 2.6-cm sterile Loach guide wire was placed through the routinely indwelling mediastinal and pericardial rubber drainage tubes during operation. (D) The indwelling pleural drainage tube and pericardial drainage tube were removed. (E) The negative pressure groove drainage tube along the guide wire was placed. (F) Diagram of the same surgical position on left side and right side.

**Fig. 2:**
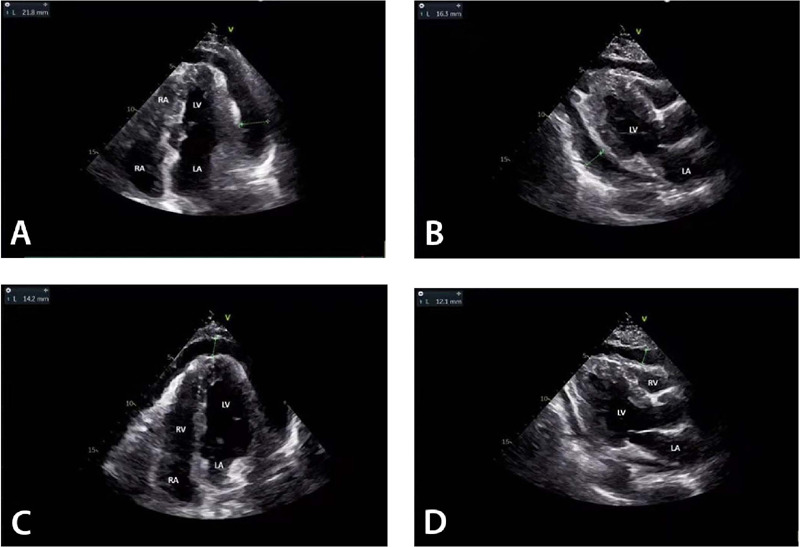
Bedside color Doppler ultrasound was performed to determine the location of effusion and/or associated pericardial thickening. The effusion was mainly located in the posterior wall of left ventricle (LV) (A), left ventricular wall (B), right ventricular wall (C), and cardiac apex (D). LA=left atrium; RA=right atrium; RV=right ventricle.

All pericardial drainages were performed with disposable sterile negative pressure groove drainage tube and guided by transthoracic color Doppler ultrasound. The coagulation profile and unsatisfactory bleeding profile were corrected before intervention in all patients. All patients were placed in supine position (Figure 1A), and then, the location of effusion and/or associated pericardial thickening was confirmed under the guidance of bedside color Doppler ultrasound by the surgeon (Figure 2). Complex iodine was used to disinfect the median sternal incision, pericardium, and mediastinal drainage tube. A conventional sterile draping was placed (Figure 1B). 0.2% lidocaine was subcutaneously injected to anesthetize the skin and subcutaneous tissues of the mediastinal and pericardial drainage orifices. A 2.6-cm sterile Loach guide wire (Terumo, Tokyo, Japan) was placed through the routinely indwelling mediastinal and pericardial rubber drainage tubes during operation (Figure 1C). The indwelling pleural drainage tube and pericardial drainage tube were removed along the guide wire (Figure 1D), and then the catheter of the negative pressure groove drainage device was placed along the guide wire (Figure 1E). The position of the drainage tube was adjusted under the guidance of color Doppler ultrasound, and then, set off to slowly drain. After the drainage tube was fixed to the skin at the initial drainage orifice by two regular stitches, the drainage bottle (Sanrui, Jiangsu, China) was connected. Complex iodine was used to disinfect the incision two times. Sterile dressing was used to cover the incision. The same procedure was performed on the opposite side (Figure 1F).

## RESULTS

From January 1, 2019 to December 31, 2021, a total of 20 patients with pericardial effusion after cardiac surgery underwent transthoracic color Doppler ultrasound-guided grooved negative pressure drainage tube implantation. The average drainage duration was 7.4±3.2 days (Table 2), and the cumulative drainage volume was 606.8±340.8 mL (Table 3). Wound infection was reported in two cases, who recovered smoothly after second stage debridement and suture. There was no recurrence of pericardial effusion. After the operation, the symptoms (chest tightness, shortness of breath, etc.) of each patient were alleviated. Postoperative color Doppler ultrasound showed the effusion had been completely drained, and then, the drainage tube was removed. No obvious complications were recorded during the operation.

**Table 2:** Summary of the operation data.

Variables	Data
Surgery, n (%)	
MVR+TVP	4 (20.0)
AVR	1 (5.0)
MVR+AVR+TVP	1 (5.0)
CABG	1 (5.0)
Wheat*+MVP+TVP	1 (5.0)
ASD	1 (5.0)
VSD+TVP	1 (5.0)
F4+PBPV	1 (5.0)
PDA+MVP+TVP	1 (5.0)
AAR	1 (5.0)
AAR+TAR+stent implantation	7 (35.0)
Total cross-clamping time (min)±SD	100.1±37.5
Total bypass time (min)±SD	174.1±60.7
Total surgery time (min)±SD	137.1±124.3
Total blooding volume (mL)±SD	450±235.1
Average recovery time (min)±SD	725.1±1445.5 (268.2±244.1)
Usage time of respirator (hours)±SD	53.8±55.0
Duration of ICU (hours)±SD	111.3±52.3
In-hospital stay (days)±SD	37.6±17.3

* Wheat procedure = preservation of the aortic valve and ascending aorta replacement

AAR=ascending aortic replacement; ASD=atrial septal defect; AVR=aortic valve replacement; CABG=coronary artery bypass grafting; F4=tetralogy of Fallot; ICU=intensive care unit; MVP=mitral valvuloplasty; MVR=mitral valve replacement; PBPV=percutaneous balloon pulmonary valvuloplasty; PDA=patent ductus arteriosus; SD=standard deviation; TAR=total arch replacement; TVP=tricuspid valvuloplasty; VSD=ventricular septal defect

**Table 3:** Summary of drainage results.

Variables	Data
Position of pericardial effusion, n (%)	
Posterior wall of left ventricle	4 (20.0)
Posterior wall of left ventricle + anterior mediastinum	3 (15.0)
Posterior wall of left ventricle + right pericardium	1 (5.0)
Posterior wall of left ventricle + anterior pericardium	4 (20.0)
Posterior wall of left ventricle + anterior mediastinum + right pericardium	4 (20.0)
Posterior wall of left ventricle + anterior pericardium + right pericardium	4 (20.0)
Total volume of pericardial effusion*, n (%)	
< 10 mm	4 (20.0)
10~20 mm	8 (40.0)
> 20 mm	8 (40.0)
Total volume of drainage after catheterization (mL)±SD	606.8±340.2
Total time of drainage after catheterization (days)±SD	7.4±3.2
Preoperative EF (%)±SD	56.1±15.5
Preoperative LVEDV (mm)±SD	43.1±11.2

* The classification of pericardial effusion was determined based on color Doppler echocardiography

EF=ejection fraction; LVEDV=left ventricular end-diastolic volume; SD=standard deviation

## DISCUSSION

At present, the diagnosis and treatment strategies of postoperative pericardial effusion are endless. With the improvement of suturing technique, of hemostasis during operation^[13]^, and of postoperative care^[14]^ and the appearance of enhanced recovery, the number of patients who need surgical intervention has been reduced. Nevertheless, postoperative pericardial effusion is reported as one of the most common complications after cardiac surgery. The causes of pericardial effusion are known and often associated with various events, such as poor drainage, excessive exudation, abnormal coagulation, incomplete hemostasis, etc. Once pericardial effusion is formed, it will not only affect the postoperative recovery and prolong the patient’s in-hospital stay, but also even threat the patient’s life.

In clinical practice, there are many ways to intervene pericardial effusion. The conservative treatment of cardiac diuresis had proved to be effective; and with the movement out of bed and follow-up of postoperative symptomatic treatment, most patients with a small to moderate amount of pericardial effusion have improved symptoms, but this also increases the duration of in-hospital stay and the associated medical costs; however, some patients with a large or moderate amount of effusion do not have improved symptoms, which causes a series of fatal cardiovascular events, such as heart failure or cardiac tamponade^[13,14,15,16,17]^, etc. Ultrasound-guided pericardiocentesis and pericardial fenestration have been clinically proved to be effective for pericardial effusion, which can relieve the symptoms immediately in the event of an emergency^[16,17]^. However, these methods also have their limitations in clinical application. For pericardiocentesis, puncture is more difficult when the effusion is located in the posterior wall of the heart^[17]^ and easy to cause arrhythmia, coronary artery or pericardium injury, hemothorax, pneumothorax, pneumopericardium, and liver injury^[1]^. When pericardial effusion is accompanied by blood clot, the risk of a failed pericardiocentesis is greatly increased. Pericardial fenestration is a complex surgical procedure to create a passage or “window” from the pericardium to the pleural cavity, allowing the pericardial fluid to drain into the pleural cavity around the heart, to prevent or treat a massive pericardium or pericardium tamponade. Although pericardial fenestration is effective for pericardial effusion, it is also massively traumatic to the patients^[5,11,13,14,15,16,17]^.

Compared with the two abovementioned methods, the transthoracic color Doppler ultrasound-guided grooved negative pressure drainage has many advantages as the emerging alternative. Firstly, it is through the original pipeline, which don’t add a new channel for drainage and is less likely to damage the heart and/or blood vessels. Secondly, it has no requirement for general anesthesia, compared with pericardial fenestration, so it is less invasive, especially for the patient who has just undergone aortic dissection surgery or those with cardiac insufficiency. In other words, this procedure is easy to perform, prompt, safe, effective, and does not increase the obvious wound, so the postoperative recovery can be obviously accelerated. Thirdly, the early and adequate drainage can effectively eliminate the cavity needed for occurrence of hydrops and prevent recurrence of hydrops or readmission of patients. Finally, early intervention of pericardial effusion before extubation can attract more doctors’ attention to make a more perfect postoperative diagnosis and a better treatment strategy, which can effectively improve patients’ symptoms and accelerate the patients’ recovery.

Before the extubation, complete cardiac color Doppler echocardiography to provide valuable information about the loculated and septate effusions can make clear the causes of pericardial effusion and formulate the corresponding diagnosis and treatment strategy, conducing to timely prevent or reduce the risk of development of pericardial effusion to cardiac tamponade, or even death. Of course, some factors are definitely correlated with the occurrence of pericardial effusion, such as intraoperative management, including adequate hemostasis, rational anticoagulation, and treatment of primary diseases (cardiac insufficiency, multiple organ failure, etc.)^[5]^, and postoperative care (checking the drainage tube regularly for patency and so on). Active treatment can prevent and/or treat pericardial effusion in the perioperative period.

This study focuses on pericardial effusion in the perioperative period, especially before removal of the drainage tube, which is conducive to early prevention, detection, and/or intervention of pericardial effusion. It is a rapid, safe, and effective procedure with non-obvious trauma, so it is of great clinical significance for postoperative rehabilitation of patients

### Limitations

First, after cardiac surgery, the incidence of massive pericardial effusion or cardiac tamponade is not high, however, once it occurs, the impact on postoperative recovery is very large, so the number of cases that our center can provide is relatively small. And second, the postoperative pericardial effusion is mostly a small or moderate amount clinically, so the selected scheme is generally conservative treatment. For a large number of patients, methods such as pericardiocentesis and effusion extraction can be selected, however, a suitable site for pericardiocentesis is required, and the vast majority of patients in our center have no indication that the operation can be performed, so there is no approach to compare them in this study.

## CONCLUSION

In our study, data obtained from 20 patients with pericardial effusion showed that the symptoms were all relieved after transthoracic color Doppler ultrasound-guided grooved negative pressure drainage tube implantation and that the drainage is unobstructed. It appears to be an effective and safe alternative for drainage of pericardial effusion with minimal invasion, shorter recovery time and in-hospital stay, and fewer complications, which is worthy of clinical promotion.

## Abbreviations, Acronyms & Symbols

AAR: Ascending aortic replacement
ASD: Atrial septal defect
AVR: Aortic valve replacement
CABG: Coronary artery bypass grafting
EF: Ejection fraction
F4: Tetralogy of Fallot
ICU: Intensive care unit
LA: Left atrium
LV: Left ventricle
LVEDV: Left ventricular end-diastolic volume
MVP: Mitral valvuloplasty
MVR: Mitral valve replacement
NYHA: New York Heart Association
PBPV: Percutaneous balloon pulmonary valvuloplasty
PDA: Patent ductus arteriosus
RA: Right atrium
RV: Right ventricle
SD: Standard deviation
TAR: Total arch replacement
TVP: Tricuspid valvuloplasty
VSD: Ventricular septal defect
